# Probiotic-derived extracellular vesicles: the next breakthrough in postbiotics for rheumatoid arthritis

**DOI:** 10.3389/fimmu.2025.1620185

**Published:** 2025-08-07

**Authors:** Federica Dell’Atti, Hugo Abreu, Patrizia Malfa, Davide Raineri, Giuseppe Cappellano, Annalisa Chiocchetti

**Affiliations:** ^1^ Department of Health Sciences, Interdisciplinary Research Center of Autoimmune Diseases (IRCAD), Università del Piemonte Orientale, Novara, Italy; ^2^ Center for Translational Research on Autoimmune and Allergic Disease (CAAD), Università del Piemonte Orientale, Novara, Italy; ^3^ R&D Department, SynBalance Srl, Origgio, VA, Italy

**Keywords:** extracellular vesicles, probiotics, dysbiosis, inflammation, arthritis, immunomodulation, oral-gut-joint axis, therapeutic strategies

## Abstract

Rheumatoid arthritis (RA) is a chronic autoimmune disease characterized by systemic inflammation and joint damage. Emerging evidence highlights the role of gut and oral microbiota in RA pathogenesis, with microbial dysbiosis potentially exacerbating inflammation and immune dysregulation. Although probiotics have shown potential in modulating the oral and gut microbiota and improving RA symptoms, a promising cell-free substitute is provided by postbiotics, including probiotic-derived extracellular vesicles (EVs). These bioactive nanoparticles transport functional metabolites capable of modulating immune responses, reducing inflammation, and restoring gut barrier integrity. Probiotic-derived EVs are, for instance, able to promote M2 macrophage polarization and suppress pro-inflammatory cytokines, thus highlighting their therapeutic potential. Nonetheless, challenges remain in standardizing EVs production, optimizing administration routes, and ensuring clinical safety. The targeting and effectiveness of probiotic EVs may be improved by developments in omics sciences and biotechnology techniques, making them the next breakthrough in postbiotics for the treatment of RA. This review examines how probiotic-derived EVs interact with the host, focusing on their crosstalk with immune cells and subsequent immune modulation. We highlight their potential for RA treatment, discuss clinical challenges, and explore their use in personalized medicine.

## 1 Introduction

Rheumatoid arthritis (RA) is a chronic autoimmune disease affecting almost 1% of the adult population and manifesting with several systemic symptoms that converge into joint inflammation leading to articular pain and decreased mobility (1, 2). RA is characterized by the inflammation of the synovium of the joints caused by the autoimmune aggression leading to the secretion of pro-inflammatory cytokines causing swelling, pain and joint damage. The persistent systemic inflammation is driven by the different types of immune cells. CD4+ T helper (Th) cells release cytokines, such as interleukin (IL)-2, IL-4 and IL-21, which are crucial for further activating other immune cells, including macrophages and B cells. Once activated, macrophages secrete mainly tumor necrosis factor-alpha (TNF-α), IL-1β and IL-6, while B cells produce autoantibodies, such as rheumatoid factor (RF) and anti-citrullinated protein antibodies (ACPAs), that further increase the systemic effects and joint damage (3–5). Interestingly, RA is influenced by a transdifferentiation of Th17 to a pathogenic phenotype of Th1 (6, 7), complicating the whole picture.

TNF-α and IL-6 contribute significantly to synovial inflammation by activating fibroblast-like synoviocytes (FLS) and macrophages, thereby establishing a self-perpetuating inflammatory loop. TNF-α also promotes the production of matrix metalloproteinases (MMPs), enzymes that degrade the extracellular matrix (ECM), leading to cartilage destruction (8). IL-6 supports B cell activation and promotes the differentiation of T cells into pathogenic Th17 ones (9). In parallel, joint erosion is driven by osteoclast activation and bone resorption, both enhanced by receptor activator of nuclear factor kappa-B ligand (RANKL), which is mainly expressed by both activated T cells and FLS (10).

Current therapies with nonsteroidal anti-inflammatory drugs (NSAIDs), disease-modifying anti-rheumatic drugs (DMARDs) and biologic agents are aimed at managing symptoms, decreasing pain and inflammation (11). For instance, NSAIDs can provide only temporary relief without affecting the cause of the disease (12). Conventional DMARDs, such as methotrexate, are sometimes associated with side effects, like general fatigue, diarrhea and even hepatotoxicity (13). Moreover, even if the biologic agents can target specific immune pathways, they may increase susceptibility to infections, and a high percentage of RA patients exhibit inadequate or no response (14).

However, do to the lack of predictive biomarkers, this therapeutic strategy relies on a trial-and-error approach, which negatively impacts the quality of life of subjects with RA, leads to development of the above mentioned collateral effects and places a significant financial burden on national healthcare systems (15, 16). Therefore, new, safe, and highly effective therapeutic strategies are needed to fulfil the unmet requirements in RA care.

Several recent findings have revealed that gut and oral microbiota play a role in the pathogenesis of RA. An imbalance in both the composition and relative abundance of microbial communities, known as dysbiosis, has been shown to contribute to systemic inflammation, potentially exacerbating disease progression (17, 18).

Among the many bacteria living in the human intestine, specific bacteria such as *Prevotella* spp., *Porphyromonas gingivalis* and *Lactobacillus* spp. showed altered abundance in patients affected by RA compared to healthy controls (HC). For instance, *Prevotella* spp. and *P. gingivalis* are increased in RA subjects, whereas the percentage of *Lactobacillus* spp. increases or decreases depending on the specific strain (19–23). These microbial imbalances contribute to increased gut permeability and systemic inflammation (24), enabling microbial antigens and metabolites to translocate into the bloodstream and potentially exacerbate RA progression. Importantly, *P. gingivalis* expresses a special enzyme called peptidylarginine deiminase (PAD) that catalyzes protein citrullination, a process which may disrupt immune tolerance and initiate the production of ACPAs (25). In addition, *Prevotella copri* and *Aggregatibacter actinomycetemcomitans* can indirectly promote hypercitrullination by enhancing host PAD activity at mucosal surfaces (26–28).

Probiotics are live microorganisms known for their health benefits (29). By modulating the gut microbiota composition, probiotics may help to restore microbial balance, to reduce inflammation, and to alleviate symptoms associated with autoimmune and inflammatory conditions, including RA (30–33). The aforementioned beneficial effects are mediated by probiotics’ metabolites, known as postbiotics.

The latest definition of postbiotics, established by the International Scientific Association for Probiotics and Prebiotics (ISAPP), characterizes them as “preparations of inanimate microorganisms and/or their components that provide health benefits to the host” (34). Recent studies suggest that bacterial extracellular vesicles (bEVs) may represent a promising next generation of postbiotics.

EVs are small membrane-bound particles secreted by both eukaryotic and bacterial cells (35, 36). EVs contain bioactive molecules, including proteins, lipids, and RNA, which can influence cellular functions and modulate immune responses of recipient cells (37, 38).

**Figure 1:**
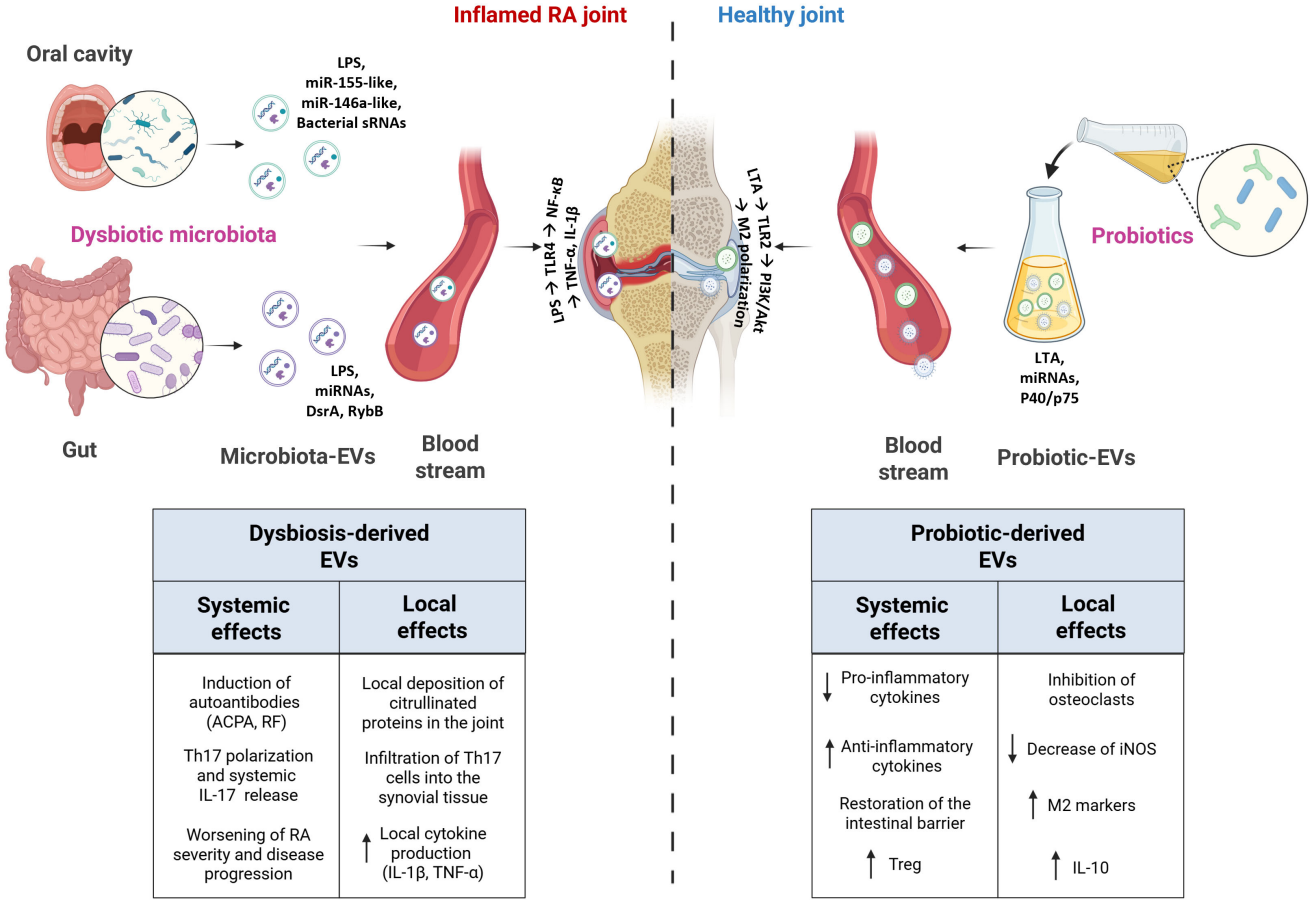
Schematic representation of the systemic and local effects of bacteria-derived EVs in RA and healthy joints. In RA, dysbiotic oral and gut microbiota release EVs that can induce systemic immune dysregulation and promote local joint inflammation, thereby exacerbating disease severity and progression. Conversely, probiotic-derived EVs exert protective effects by reducing pro-inflammatory cytokine production, inhibiting osteoclast activation, and restoring intestinal barrier integrity.

**Figure 2:**
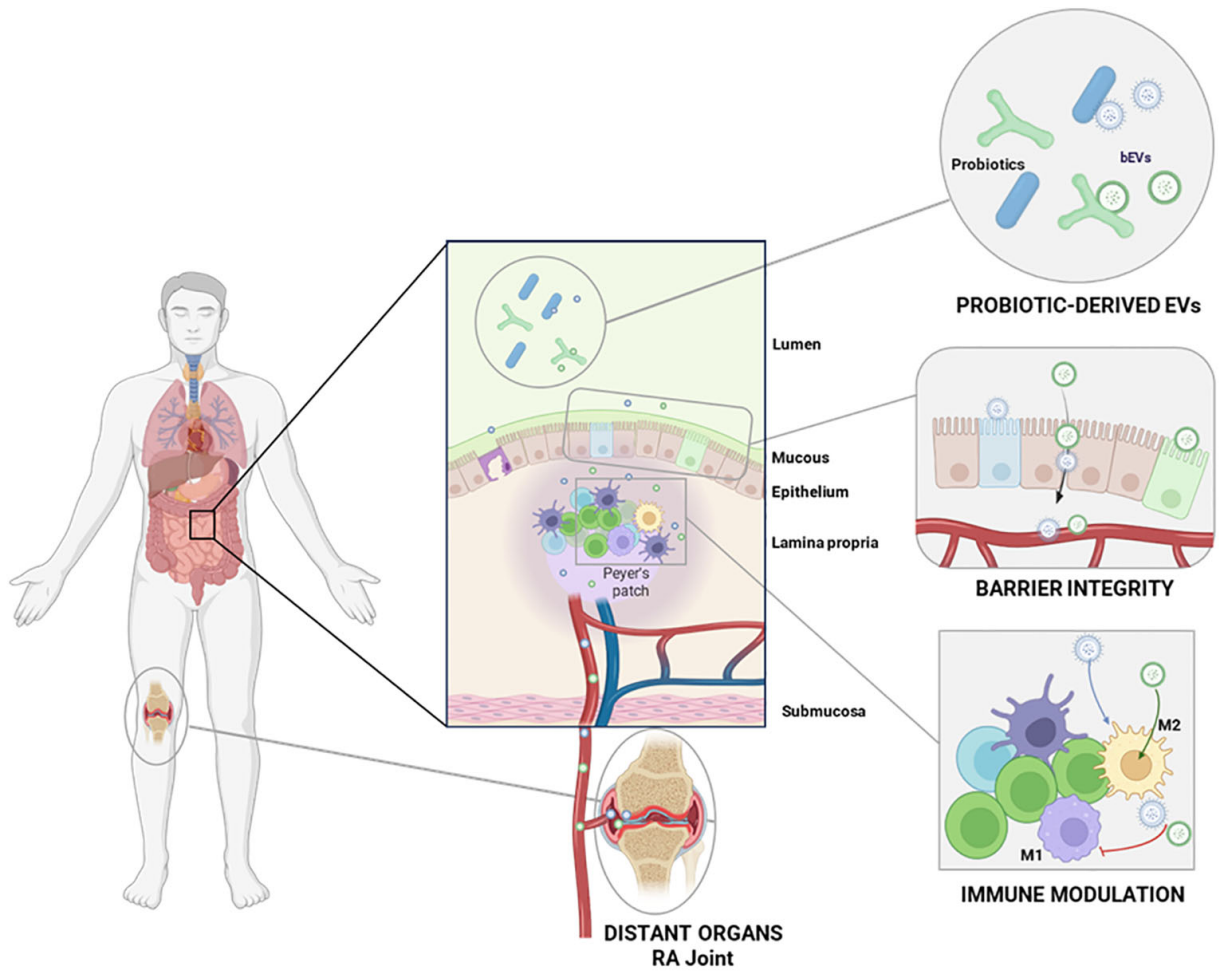
Overview of the probiotic-derived EVs interactions with the host mucosa, with the immune system and distant organs. Probiotic-derived EVs interact with the intestinal epithelium and Peyer’s patches, modulating the local immune response and the intestinal barrier integrity. Upon entering circulation, these EVs can also reach distant organs such as inflamed RA joints.

The driving hypothesis of this review is that probiotics might exert their therapeutic effects through the release of EVs that in turn might interact with the immune system, influencing immune cell function, modulating inflammatory responses (39) and antimicrobial effects in distant districts (40) (**Figure 1**). Probiotic-derived EVs might interact with the intestinal epithelium and Peyer’s patches influencing the immune modulation. The bioactive metabolites carried by bEVs may also enhance gut barrier integrity, by promoting intestinal health and preventing the leakage of harmful pathogens or toxins into the bloodstream, which can exacerbate systemic inflammation (**Figure 2**).

The ability of probiotics to release such metabolites through EVs offers a promising mechanism for their therapeutic action, potentially leading to new, more targeted strategies for treating inflammatory and autoimmune conditions. This approach could also reduce the need for traditional pharmaceuticals, providing a more natural and less toxic alternative for managing diseases linked to immune dysregulation.

To address the topic, this review explores how probiotic-derived EVs interact with the host, focusing on their crosstalk with immune cells and the underlying mechanisms that modulate immune responses. By delving into their immune-modulatory and inflammation-suppressing properties, we seek to uncover how probiotic EVs could offer a novel strategy for RA treatment. Additionally, we will address the current obstacles and limitations of clinical translation for probiotic-derived EVs particularly in the context of their use in clinical settings. Furthermore, we will explore the possibility of using these EVs as a therapy in a patient-tailored approach.

## 2 Gut and oral microbiota dysbiosis in RA

It has been widely demonstrated that gut and oral microbiota have an important role in the pathogenesis of RA. It is still unclear whether the condition of dysbiosis is the cause of the disease or the consequence of the chronic systemic inflammation, but there is strong clinical evidence of bacteria involvement in this disease (19, 41).

Gut and oral microbiota can play a causal role in the development of RA, especially by favoring an environment that can lead to autoreactive immune responses. Some pathogenic oral microorganisms, such as *Porphyromonas gingivalis* and *A. actinomycetemcomitans*, and gut bacteria, such as *P. copri*, can enhance the generation of citrullinated proteins (26, 42, 43). *P. gingivalis* expresses the peptidylarginine deiminase (PAD) enzyme that directly citrullinates host proteins (25), while *A. actinomycetemcomitans* and *P. copri* can indirectly induce hypercitrullination by increasing host PAD activity in the oral and gut mucosa respectively (26–28). These citrullinated proteins generated by both microbial and inflammation-driven mechanisms can facilitate the ACPAs production which is a hallmark of RA pathogenesis (44). Regarding the dysbiosis as a consequence of RA, on one side, metagenomic studies showed that subjects with RA have a different microbiota composition compared to HC, in terms of both abundance and diversity of the bacterial populations. Among the bacterial species that have been identified in fecal samples of patients, *P. copri*, *Akkermansia* and *Coprococcus* species, and *Ruminococcus gnavus* have exhibited an enhanced abundance, while *Haemophilus*, *Bacteroides*, and *Bifidobacterium* were significantly decreased (45, 46).

*P. copri* is present in the 75% of new-onset RA patients, compared to only 21,4% in HC (45, 47). In addition, *Ruminococcus gnavus* was found enriched in young RA patients (48), while *Bifidobacterium* genera was correlated with decreased levels of RF and C-reactive protein (CRP) (46, 49). *Akkermansia* and *Coprococcus* genera have also shown an increased abundance, though their functional roles remain less clear (50, 51).

An increase of *P. copri* has been associated with inflammation and induction of Th17 inflammation pattern and production and circulation of autoantibodies, possibly suggesting its involvement in RA pathogenesis (52). Instead, the increase of *R. gnavus* has been linked to an altered cytokine production, such as IL-1β, and degradation of mucin, essential for the gastrointestinal barrier integrity (45, 53, 54). Additionally, different clinical trials have confirmed an increased presence of *Prevotella* spp., particularly *P. copri*, in feces of early-stage RA. It has been postulated that *P. copri* triggers the activation of Th17 with the consequent production of IL-17, which play a key role in the pathogenesis of RA (55, 56). Another study showed that *Mediterraneibacter tenuis* and *Eubacterium rectale* have been associated with lower levels of IL-10, an anti-inflammatory cytokine, as well as a positive correlation with other clinical parameters such as the erythrocyte sedimentation rate (ESR), a marker of systemic inflammation. These findings possibly suggest that these bacterial species may play a role in modulating the inflammatory processes associated with RA. By influencing the production of IL-10, which normally helps to regulate inflammation, *M. tenuis* and *E. rectale* may contribute to the imbalance between pro-inflammatory and anti-inflammatory responses seen in RA (46). Conversely, decreased abundance of the genera *Bacteroides* and *Bifidobacterium*, which are very important in maintaining gut health, might contribute to dysbiosis and increased inflammation (45, 57).

On the other side, preclinical studies done in mice have shown that restoration of gut homeostasis is associated with arthritic score amelioration (58). In addition, fecal microbiota transplantation (FMT) of microbiota derived by RA patients in germ-free arthritis-prone SKG mice resulted in development of Th17 response associated to severe arthritis (59).

Regarding the mechanism by which gut dysbiosis promotes systemic inflammation and RA pathogenesis, one key aspect is the reduced abundance of short-chain fatty acid (SCFA)-producing bacteria, such as *Faecalibacterium prausnitzii* (60), that lead to an impaired intestinal barrier function and to a decrease in the activation of regulatory T cell (Treg) and an increase in pro-inflammatory Th17 response (61). A deficiency in these bacteria contributes to an increased intestinal permeability, known as “leaky gut”, which allows microbial metabolites and components such as lipopolysaccharides (LPS) to translocate into the circulation and trigger chronic inflammation. Supporting this, Heidt et al. found elevated levels of fecal zonulin, a biomarker of gut permeability, in 98% of patients with early-stage RA, underscoring the association between barrier dysfunction and disease onset (62).

Furthermore, dysbiosis disrupts also bile acids metabolism by decreasing the production of primary bile acids such as cholic, deoxycholic and lithocholic acid. These bile acids not only showed anti-inflammatory properties in *in vivo* RA model (63), but can also regulate bone metabolism through the activation of the bile acid membrane receptor TGR5, which decreases osteoclast differentiation both in *in vitro* and *in vivo* settings (64). Notably, expression of TGR5 mRNA in peripheral blood mononuclear cells (PBMCs) from RA patients was significantly lower than in HC, suggesting a link between dysregulated bile acid signaling and bone loss in RA (63).

Recent findings suggest that an imbalance in the oral microbiota might also play a pivotal role in the pathogenesis of RA. In RA patients, the oral microbiota is characterized by an increased abundance of pathogenic bacteria such as *P. gingivalis*, *A. actinomycetemcomitans*, and *P. copri* (52, 65). Notably, *P. gingivalis* has been shown to be significantly more abundant in the tongue biofilm of RA patients, with 48,9% of RA patients tested positive compared to 33,3% of HC (66). Patients with established RA are shown to be more prone to have *A. actinomycetemcomitans* infections, affecting approximately 47% of individuals (27). Conversely, beneficial commensal bacteria such *Ligilactobacillus salivarius* are decreased (52). The implications of these shifts extend beyond dysbiosis since they have been shown to modulate autoimmune responses. For example, it has been demonstrated that pathogenic *Streptococcus* species isolated from oral swabs of RA subjects worsen the severity of arthritis in experimental mouse models (67, 68). Moreover, *Prevotella*, *Leptotrichia* and *Neisseria* oral taxa correlate with serum levels of ACPA and RF, indicating a potential biomarker role for disease activity and progression (69). Compared to healthy individuals, patients with new-onset RA (NORA) exhibited a distinctive composition of the subgingival microbiota, characterized by higher abundance of some bacterial genera, including *Anaeroglobus*, *Prevotella*, and *Leptotrichia* (70). In particular, *Prevotella* and *Lepotrichia* species were absent from the oral microbiota of HC and were observed only in NORA patients, with prevalences of 32,2% and 25,8%, respectively (71). These findings support the hypothesis that specific oral microbiota components may contribute to RA onset and serve as early indicators of disease.

Noteworthy, specific bacterial taxa in the oral microbiota, such as *P. gingivalis*, have been linked to RA progression and onset, but also with periodontal disease (PD) which may act as a trigger for systemic autoimmune processes (72). More than 75% of RA patients had moderate to severe PD, underscoring a strong correlation between the severity of PD and the presence of RA. This association suggests that PD may not only be a common comorbidity in RA patients but could also play a role in exacerbating the systemic inflammation, highlighting the importance of maintaining oral health and addressing PD as part of a comprehensive strategy for managing RA, potentially reducing inflammation and improving disease outcomes (73). However, not all periodontal pathogens, including *P. gingivalis*, are uniquely associated with RA onset, suggesting a more complex interplay between oral health and systemic diseases.

All these evidences support that dysbiosis in the gut and oral microbiota contributes to the development and progression of RA, influencing systemic inflammation and immune regulation (74).

Although this imbalance of microbial communities in RA, certain bacteria, such as the probiotic *Lacticaseibacillus casei*, may have beneficial effects in reducing pro-inflammatory cytokines by inhibiting COX-2 and NF-kB, which are enzymes known to mediate inflammatory processes associated with RA. This suggests a complex and potentially beneficial relationship between gut microbiota and RA management (52).

## 3 Probiotics beneficial effects

Probiotics can exert their beneficial effects on the host through several mechanisms including the production of metabolites, maintenance of the intestinal barrier integrity, and immunomodulation. Due to their content and strain specific effects, probiotics should be selected carefully based on the disease mechanisms (75) as a strain effective for one condition may be ineffective or even detrimental in another. For instance, some adverse effects have also been proposed, such as diarrhea, nausea or other deleterious immunological effects based on molecular mimicry (76, 77).

Metabolites, such as short-chain fatty acids (SCFAs), organic acids, tryptophan-related compounds, and bacteriocins, interact with the host specific receptors, mainly G-protein coupled receptors (78), and influence immune responses (79–81). For instance, *Lactocaseibacillus casei* has been shown to increase the production of SCFAs, particularly butyrate, which are linked to an enhanced regulatory T cell (Treg) activity. This, in turn, might potentially reduce the inflammation in RA (82). Eicosapentaenoic and docosapentaenoic acids are modulated by probiotics and are known to possess anti-inflammatory properties (82). These metabolites are also essential to support the intestinal barrier integrity since they are able to mediate the expression of tight junctions and increase the mucin secretion by goblet cells (83–85).

Probiotics also modulate the Toll-like receptor (TLR) signaling pathways (86) that are known to play an important role in immune modulation. In particular, Gram-negative (Gram−) strains interact with TLR4, while Gram- positive (Gram +) ones through TLR2 (87). Specifically, probiotics can attenuate TLR signaling by upregulating Toll interacting protein (Tollip) and Interleukin-1 receptor-associated kinase M (IRAK-M). These molecules inhibit downstream signaling cascades, thereby preventing NF-κB activation and reducing the production of pro-inflammatory cytokines (88). Moreover, activation of Toll-like receptor 2 (TLR2) can induce the relocalization (or redistribution) of tight junction proteins, including zonular-occludins 1, which are essential for preserving the gut’s selective permeability (89–92). These beneficial microorganisms can therefore modulate both innate and adaptive immunity by interacting with T cells, dendritic cells and macrophages. Probiotics often enhance the secretion of anti-inflammatory cytokines such as IL-10 and transforming growth factor beta (TGF-β) while simultaneously reducing levels of pro-inflammatory cytokines such as TNF-α, IL-6, and IL-17 (93–97).

Furthermore, probiotics have been also associated with the modulation of the production of nitric oxide metabolites, indicating that they are able to induce a decrease in oxidative stress (95, 98), which is often elevated in RA patients, leading to tissue damage and the progression of the disease.

Interestingly, probiotics and their postbiotics can also have an impact on trained immunity, which is a recently described type of innate immune memory characterized by long-term epigenetic and metabolic reprogramming, leading to enhanced immune responses to secondary stimuli (99, 100). While trained immunity serves as an adaptive mechanism that strengthens the innate immune response against secondary infections, it can also contribute to excessive inflammation in autoimmune diseases. Thus, modulating trained immunity offers a promising therapeutic approach for immune-related disorders (99). Specifically, *L. plantarum* was used to successfully prime monocytes and macrophages to develop an innate immune memory, by promoting anti-inflammatory mechanisms, such as increased IL-10 production, and altering the cells’ transcriptomic and metabolic profile. In fact, a downregulation of several pro-inflammatory molecules, such as TNF-α and IL-1β was detected, together with a lower reactive oxygen species (ROS) production, upon the administration of a second stimulus (93). Remarkably, *Limosilactobacillus reuteri* DSM 17938 is able to induce a memory-like phenotype in human dendritic cells that initially produce elevated levels of IL-6 and IL-1β, but upon secondary stimulation with probiotic metabolites, produce reduced levels of TNF-α and IL-23 (94). These results suggest that probiotics and postbiotics can have a quite promising role in boosting the host’s immune system response, which can be beneficial to modulate the chronic inflammation in RA. Lastly, probiotic supplementation has been linked to a reduction in CRP levels and improvements in disease activity scores for RA and axial spondyloarthritis (SpA), even if the impact of these improvements can vary among patients and the strains used (101).

Although clinical trials investigating probiotic supplementation in RA patients remain limited, several studies have reported promising outcome. These include reduction in inflammatory markers, decreasing in oxidative stress, and improvements in pain or physical function (95, 96, 102–105). Thanks to these mechanisms, probiotic bacteria may have protective and therapeutic potential to manage RA and other autoimmune disorders.

The beneficial effects of probiotics have been observed in various types of arthritis beyond RA. For instance, in patients with SpA, probiotic supplementation has been shown to increase the proportion of Tregs and to shift cytokine production toward an anti-inflammatory profile (106). In an RA animal model the administration of *Latilactobacillus sakei* induced a shift in Th17 population towards an anti-inflammatory phenotype associated to the suppression of osteoclastogenesis (107). Another study showed that probiotics decreased pro-inflammatory cytokines, such as TNF-α and IL-6, while increasing anti-inflammatory ones such as IL-10 (95–97).

Another possible way by which probiotics can exert their effects regards molecular mimicry (83, 84). This process usually occurs when the immune system mistakenly targets the body’s own tissues due to similarities between microbial and host antigens. It has been demonstrated that HLADRB1*0401, typically expressed in subject with severe RA bears a peptide similar to a heat shock protein DnaJ of the pathogenic *Escherichia coli* (108). From a protective point of view, this process theoretically might involve probiotics mimicking host molecules, which can potentially have a beneficial effect by triggering a Treg, therefore decreasing the inflammation in RA patients (109). For instance, *Bacteroides fragilis* Polysaccharide A might mimic the structure of mucin type O-glycans of the intestinal mucosa of the host thanks to the unique electrostatic structural properties, therefore modulating the intestinal barrier and promoting immune balance (110–113).

The soluble mediators addressed in this chapter are individual molecules capable of exerting discrete biological functions. However, the regulation of complex phenomena, such as disease pathogenesis or host-microbe interactions, likely requires the synergistic action of multiple factors. EVs may play a pivotal role in this context by serving as delivery vehicles for a broad spectrum of functional biomolecules, thereby orchestrating multifaceted responses in the host.

## 4 Probiotic-derived EVs

Bioactive molecules carried by probiotics can be delivered to distant tissues and organs, including joints, by EVs. Probiotic-derived EVs carry a heterogeneous cargo of metabolites, but also proteins, lipids, and nucleic acids. They are generated through specific biogenesis pathways that vary based on whether the strain is Gram+ or Gram− and play a crucial role in cell-to-cell communication (114). The cargo and the membrane components, based on the bacterial type, such as lipopolysaccharides (LPS) for Gram−, lipoteichoic acid (LTA) for Gram+, or other proteins, are important for bacterial colonization, biofilm formation, and modulation of the host immune response (115). Different factors can influence the quantity and composition of probiotic EVs, such as stress conditions, nutrient availability, and interactions with the host. Nevertheless, they retain the potential to be released into the extracellular environment (116).

### 4.1 Gram+ probiotic-derived EVs

Most probiotics are Gram+ bacteria, with the most commonly commercialized strains belonging to lactic acid bacteria (LAB) and *Bifidobacterium* genus (117), which are also the most abundant in a eubiotic microbiota. However, other microorganisms falling under this classification include various species of probiotics including *Bacillus* spp., *Propionibacterium freudenreichii*, and *F. prausnitzii*. Gram+ probiotics lack an outer membrane compared to Gram− bacteria. Given the unique composition of Gram+ bacteria wall, primarily composed of a thick peptidoglycan layer, it was initially believed they were incapable to release EVs, which has now been proved otherwise. EVs produced by *Lacticaseibacillus paracasei* exhibit variability in terms of quantity and size depending on the strains (36). *Lactiplantibacillus plantarum* Q7, *Bifidobacterium longum* AO44, and *F. prausnitzii* A2–165 have been shown to release EVs ranging from 30 to 500 nm in size (118–121). Instead, *P. freudenreichii* CIRM-BIA 129 generated EVs characterized by a distinctive spherical cup-shaped morphology, but with a diameter of 85 nm, consistent with the typical diameter range of bEVs (122).

**Figure 3:**
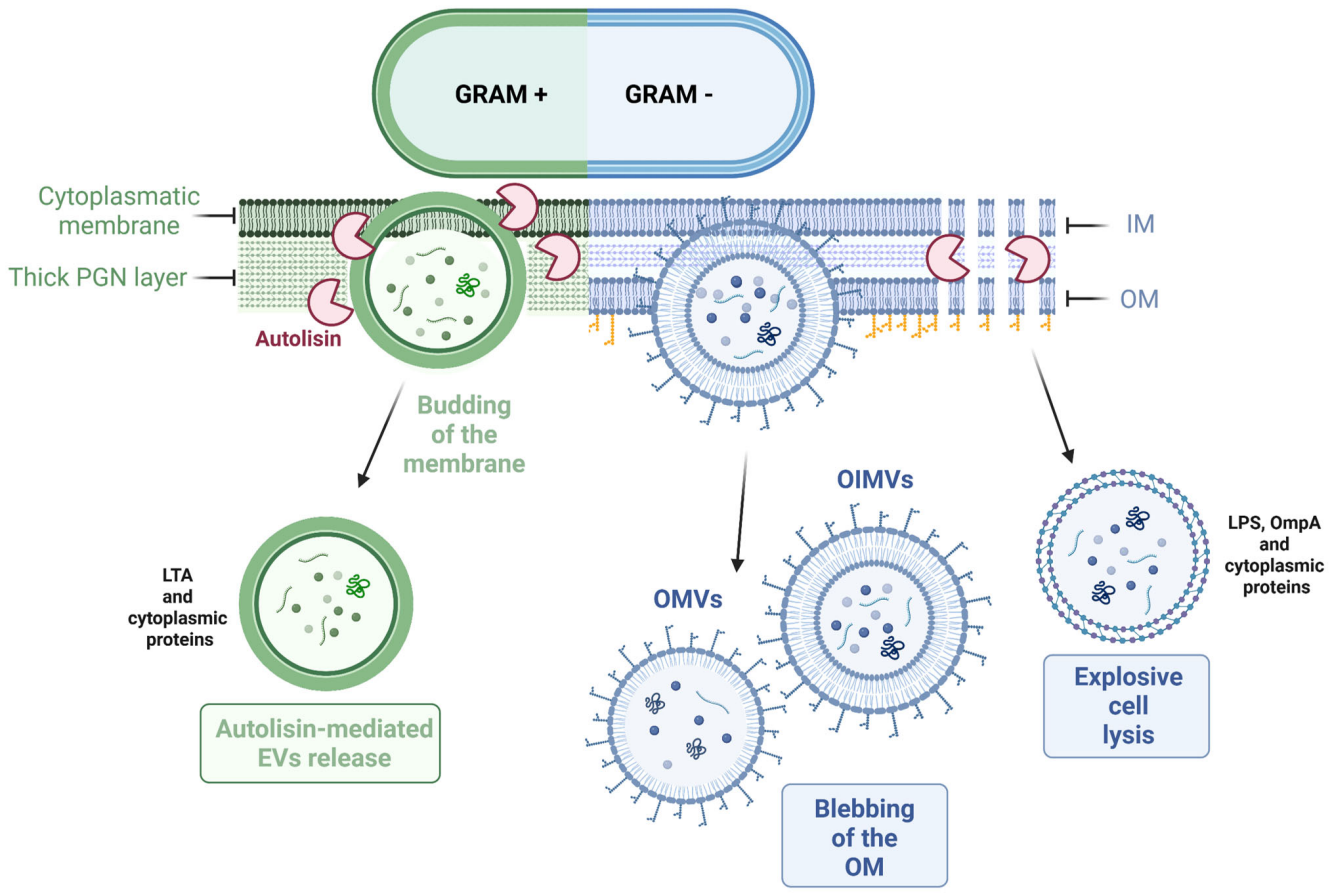
Mechanisms of EVs release in Gram+ and Gram- bacteria. Gram+ bacteria mainly release EVs through active budding of the cytoplasmic membrane. On the other hand, Gram- bacteria can produce EVs via membrane blebbing or through explosive cell lysis, which releases vesicles that contain components from the inner and/or outer membrane.

The precise mechanism of biogenesis is also thought to be species-specific. Different bacterial species, and even different strains within a species, may produce EVs through distinct pathways, influenced by factors such as their cell wall composition, growth conditions, and metabolic activity (123). The current hypothesis is that the biogenesis of these bacterial EVs might occur in two steps: the budding of the cytoplasmic membrane and the subsequent release of EVs facilitated by cell wall-modifying enzymes (**Figure 3**). The initial budding process is initiated by turgor pressure and depends on the thickness of the cell wall, which may influence the size of EVs. The release of EVs into the extracellular environment is mediated by protein involved in cell wall synthesis and remodeling, including autolysins, that might facilitate the opening of the pore in the peptidoglycan (PGN) wall, thereby promoting the release of EVs (124–126). However, the identification and characterization of specific proteins responsible for initiating EV biogenesis in Gram+ probiotics is still under investigation.

### 4.2 Gram− probiotic EVs

Gram− bacteria are less commonly used as probiotics compared to Gram+ bacteria. However, *E. coli Nissle* 1917, *Akkermansia muciniphila* and some *Paracoccus* species are used for their beneficial effects. Indeed, these probiotics can release EVs with anti-inflammatory and immunomodulatory properties (127–130). Gram− bacteria possess an outer membrane (OM) that surrounds the cell wall. This OM has an asymmetric composition, with an internal layer composed of phospholipids and an external layer enriched in LPS commonly referred to as endotoxin (131, 132). Because of this peculiar composition, these bacteria produce specific types of EVs called outer membrane vesicles (OMVs) and inner membrane vesicles (IMVs), based on the originating layer (133) (**Figure 3**). Furthermore, studies have shown that certain Gram− bacteria can release outer-inner membrane vesicles (OIMVs), which are characterized by a double-bilayer membrane (114, 134). These EVs have a size typically falling within the range of 50–250 nm. They contain various molecules, including the common constituents found in all EVs, such as PGN, as well as specific Gram− bacterial molecules such as LPS or outer membrane proteins in OMVs (135). On the other hand, IMVs and OIMVs contain cytoplasmic contents, DNA or RNA fragments, being more similar to Gram+ EVs (136, 137).

Gram− EVs biogenesis follows two pathways: blebbing of the OM and explosive cell lysis (136) (**Figure 3**). Different mechanisms have been proposed to explain the production and regulation of OMVs. Nevertheless, a conclusive mechanism of EVs biogenesis remains to be fully elucidated (132). The current hypothesis suggests that the EVs biogenesis is based on the presence of LPS, PGN, and lipoproteins (138). The first mechanism proposes that the release of EVs occurs when there are low numbers of lipoproteins associated with the PGN layer, resulting in membrane blebbing. Another blebbing strategy, specific to OMVs is based on the electric charge of LPS, influencing the formation and release of these vesicles. Lastly, the second type of mechanism involves autolysins, which break down cell wall components and facilitate a process similar to the biogenesis of Gram+ EVs (139).

### 4.3 Probiotics-derived EV cargo and immunomodulatory properties

Probiotic EVs carry a diverse range of bioactive compounds through which they can elicit therapeutic effects analogous to those exerted by the whole microorganism. In more detail, these EVs typically carry a complex cargo, which includes membrane and cytoplasmic proteins, enzymes, lipids, metabolites and even small RNAs or DNA fragments. While the precise molecular mechanisms underlying their bioactivity remain to be fully elucidated, proteomic and metabolomic analyses have revealed that these vesicles are rich in bioactive molecules that play significant roles in host-microbe interactions. Lactic acid bEVs are rich in glyceraldehyde-3-phosphate dehydrogenase (GAPDH) and enolase, involved in anti-inflammatory and pathogen response, respectively (140, 141). For instance, EVs from *L. plantarum* are enriched with enzymes involved in central metabolic pathways, such as glycolysis, and contain membrane components like amino acid transporters, which are crucial for cellular uptake and metabolic processes (142). Additionally, lipidomic studies have shown that the lipid composition of *L. plantarum*-derived EVs differs significantly from their parent cells, with certain lipid species like lysophosphatidylserine and phosphatidylcholine being highly enriched, suggesting a specialized role in intercellular communication (143). On the other hand, *L. paracasei* EVs are known to carry proteins such as P40 and P75, which are associated with immune modulation and have been shown to stimulate the epidermal growth factor receptor (EGFR) pathway, potentially contributing to gut health and epithelial protection (36, 144).

As live probiotics, EVs can modulate immune responses by influencing the balance between pro-inflammatory and anti-inflammatory cytokines. For example, *L. plantarum* UJS001-derived EVs repaired the intestinal barrier and promoted M2 polarization, increasing the production of anti-inflammatory cytokines while reducing the pro-inflammatory ones in an ulcerative colitis mouse model (145). *L. plantarum* Q7-EVs, administered in drinking water, ameliorated colitis in C57BL/6J mice by restoring the cytokine expression and regulating the gut microbiota (118). *L. plantarum* APsulloc 331261-EVs, instead, are also able to stimulate the secretion of IL-10, IL-1β and granulocyte-macrophage colony-stimulating factor (GM-CSF) in human skin organ cultures *in vitro* (119). Hence, *L. plantarum*-EVs have been shown to modulate gut homeostasis and macrophage polarization toward M2 phenotype, offering therapeutic potential by promoting anti-inflammatory responses and restoring the intestinal barrier.

*L. paracasei*-derived EVs have demonstrated anti-inflammatory properties, particularly in skin and intestinal inflammation, by augmenting the endoplasmic reticulum stress pathway and reducing pro-inflammatory cytokine levels (146, 147). *A. muciniphila* is known for its role in maintaining gut health, although specific studies on its EVs are less documented. Other probiotics such as *Lactocaseibacillus rhamnosus, L. casei, B. longum, Bifidobacterium breve, Streptococcus thermophilus, and Saccharomyces boulardii* also produce EVs that modulate immune responses and maintain the balance of the gut microbiota by inducing tolerogenic DCs and an increase in Tregs in Balb/c mice (143, 148, 149).

Further evidences showed that probiotic-derived EVs modulate the immune response by reducing pro-inflammatory cytokines (TNF-α, IL-1β, IL-6) while inducing anti-inflammatory mediators (IL-10, TGF-β) (122, 150, 151). Behind the effects on the immune system, EVs may regulate osteoclast activity, impacting bone metabolism, thereby providing potential benefits for arthritis (152). Nevertheless, even if the use of probiotics has been recorded to be safe in the majority of cases, it has been reported that in some vulnerable populations, such as the elderly and immunocompromised patients, they might have severe side effects (152). For example, parenterally administered Gram+ bacteria, by their cell wall peptide-glycanpolysaccharides, can induce fever and exacerbate arthritis and autoimmune diseases (153). Some other effects include systemic infections caused by dysbiosis and excessive immune stimulation (154). This makes probiotic EVs a potentially safer and more effective alternative to conventional probiotic treatments since they can deliver bioactive compounds without the risks associated with administration of live bacteria (148).

### 4.4 Isolation and characterization of probiotic-derived EVs

As well as all the other bEVs, EVs derived from probiotics are more frequently isolated from cell-free culture supernatants (155) using a combination of centrifugation and filtration steps. Common isolation techniquesinclude ultracentrifugation, size-exclusion chromatography (SEC), tangential flow filtration (TFF) and ultrafiltration (156). Characterization of EVs involves multiple approaches: nanoparticle tracking analysis (NTA) and dynamic light scattering (DLS) are used to measure EV concentration and size distribution, while morphology is assessed through electron microscopy techniques such as scanning electron microscopy (SEM), transmission electron microscopy (TEM), and cryo-electron microscopy (cryo-EM). To investigate their surface markers and cargo, western blotting is employed to detect specific bacterial membrane markers, such as lipoteichoic acid (LTA) in Gram + strains or LPS in Gram - species. Proteomic and metabolomic analyses are increasingly utilized to gain deeper insights into EV composition and function. Taken together, these methodologies can provide a solid characterization of probiotic-derived EVs, to ensure reproducibility and their biological effects (157). Despite these advances, purification of probiotic-derived EVs remains challenging due to contamination with proteins and cellular debris that can interfere with accurate characterization. Methods such as ultrafiltration and SEC have proven effective in improving EV yield and purity (158). However, the lack of standardized protocols and quality control procedures limits the reproducibility and broader applications of probiotic EVs.

## 5 Evidence supporting the application of probiotic-derived EVs in preclinical models of RA

As previously outlined, chronic inflammation in RA is driven by an imbalance between pro- and anti-inflammatory cytokines, as well as by the dysregulated activation and polarization of macrophages. Specifically, the pro-inflammatory milieu is predominantly sustained by classically activated M1 macrophages, which secrete a range of inflammatory mediators and ROS, and exhibit elevated activity of inducible nitric oxide synthase (iNOS) (159). These key immunopathological features collectively contribute to increased production of pro-inflammatory cytokines such as TNF-α, IL-1β, IL-6, and IL-17. The resulting cytokine cascade promotes immune cell infiltration into the arthritic joint, ultimately driving synovial hyperplasia and progressive destruction of periarticular tissues (160, 161).

This dysregulated M1/M2 ratio in RA impairs the resolution of the disease and so it is crucial to study new therapeutic approaches to restore immune homeostasis, such as probiotic EVs. Despite the lack of clinical research on probiotic EVs specific to the RA context, *in vitro* studies and investigations in preclinical models have demonstrated their potential to modulate immune responses and suppress inflammation, highlighting their promise as therapeutic agents for RA.

For instance, Woo et al. showed that EVs derived from *P. freudenreichii* (PFEVs) inhibited differentiation and activity of osteoclasts, by reducing TRAP-positive multinucleated cells and by lowering the expression of osteoclast-related genes *in vitro* (162). Furthermore, the treatment of LPS-stimulated RAW264.7 cells with *L. plantarum* (LP25)-derived EVs increases the M2 typical markers Arg-1 and IL-10 expression while reducing TNF-α and iNOS, which are associated with pro-inflammatory responses (163). These findings were then confirmed in the collagen-induced arthritis mouse model, where PFEVs treatment decreased arthritis scores and altered serum levels of proinflammatory cytokines, such as IL-6 and TNF-α (162). Another study showed that EVs derived from *Lactobacillus johnsonii* (LJEVs) injected intraperitoneally significantly mitigated arthritis progression in collagenase-induced osteoarthritis mice. In this study M1-like polarization of macrophages was inhibited and the secretion of pro-inflammatory mediators involving the glutamine synthetase/mTORC1 axis was reduced (164).

**Figure 4:**
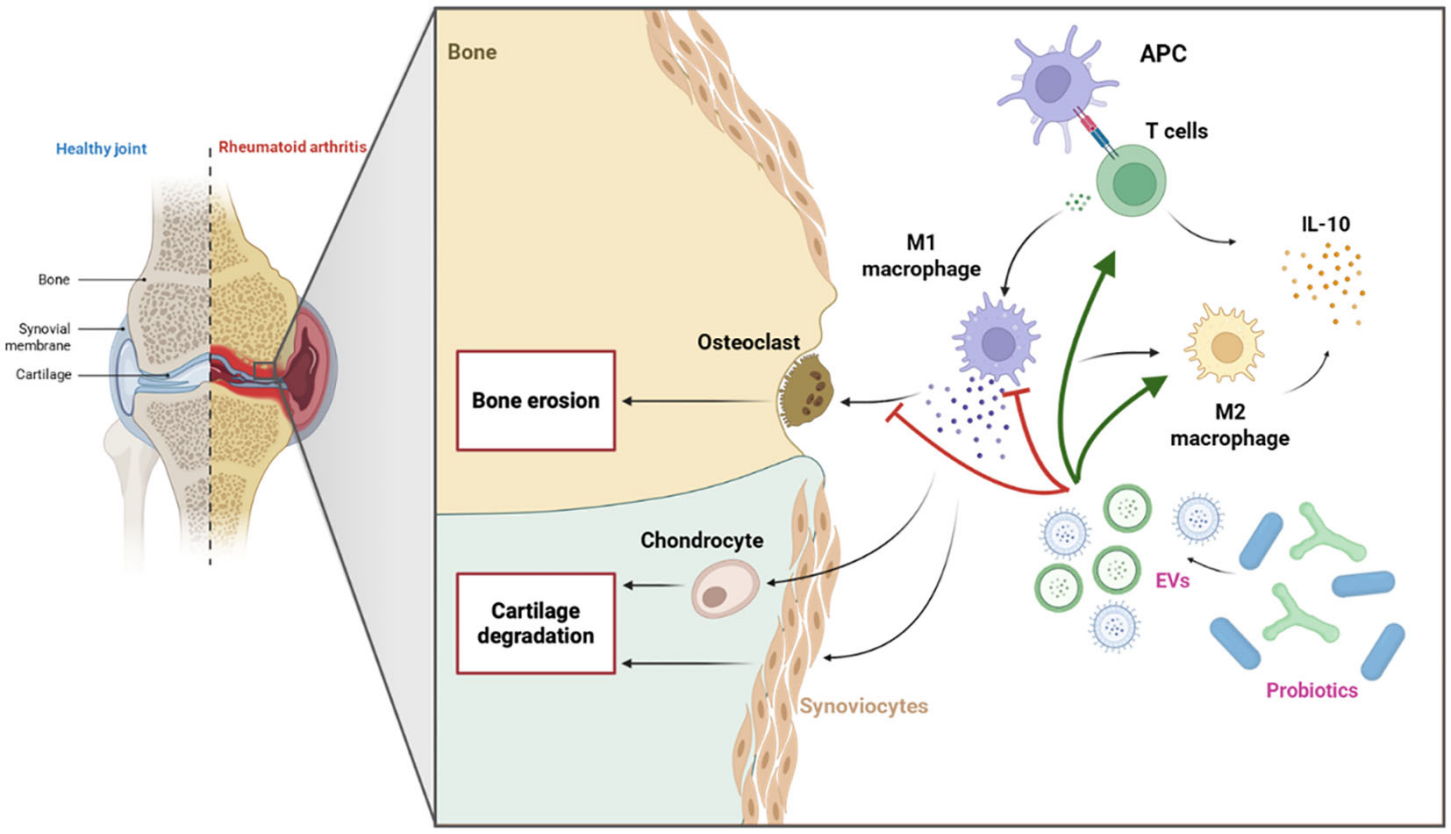
Immunomodulatory effects of probiotic-derived EVs in RA. Probiotic-derived EVs may suppress pro-inflammatory M1 macrophages and osteoclast activity, while promoting a shift toward anti-inflammatory M2 macrophages and enhancing T cell responses. Both M2 macrophages and T cells contribute to increased IL-10 secretion, collectively supporting inflammation resolution and bone protection in RA.

In summary, probiotics appear to modulate the immune response (**Figure 4**), potentially by promoting a shift toward a higher M2/M1 macrophage ratio and increasing IL-10 levels, both of which are critical for enhancing immune regulation and reducing synovial inflammation and cartilage degradation in arthritic joints (165).

Noteworthy, while traditional preclinical models, including the above-mentioned 2D *in vitro* cultures and animal models, have been instrumental in advancing our understanding of disease mechanisms, their translational relevance is limited by oversimplified architecture and interspecies differences. Unfortunately, the absence of advanced *in vitro* models for RA, such as organ-on-chip (OoC) platforms, significantly limits our ability to explore these mechanisms in a physiologically relevant context. OoC models offer a promising solution by enabling the recreation of key aspects of RA pathophysiology using patient-derived cells (166), thus providing a more accurate and human-relevant preclinical tool to investigate the immunomodulatory potential of probiotics in RA. In a human skin organoid-like culture model *L. plantarum* EVs (LPEVs) suppressed the secretion of inflammatory cytokines while increasing IL-10. Moreover, *in vitro* treatment of THP1 cells with LPEVs induced the release of M2b macrophage-specific chemokines as CXCL-1, -2, and -3 while increasing the secretion of IL-10. On the other hand, LPEVs did not have a significative impact on M1 markers, such as HLA-DRα, TLR-2 and CD68 (119). Moreover, Kang et al. created a gut-liver axis chip composed by two separate chambers – one for gut epithelial cells and another for HepG2 liver spheroids – separated by a porous membrane that allows only the transfer of soluble factors and EVs from the gut to the liver compartment. To study the effect of probiotic-derived EVs on liver tissue, HepG2 spheroids were exposed to various concentrations of *L. paracasei* and *L. casei*-derived EVs, which did not exert any toxic effect while maintaining liver function through the stimulation of albumin and urea secretion, compared with the EV-free control. As concluded by the authors, this gut-liver-on-chip model represents an important first step to study the interaction of probiotic EVs with the host’s cells as well as their therapeutic potential in a controlled organ-on-chip environment (167). In this context, the development of a joint-on-chip model would further expand the applicability of such platforms, enabling the investigation of probiotic EV effects in RA.

## 6 From bench to bedside: probiotic EVs in personalized medicine

The gut and oral microbiota are both involved in the pathophysiology of RA, as suggested by the current literature. An imbalance in certain bacterial populations in the gut or oral cavity can lead to dysbiosis, which might be both a cause and a consequence of a dysregulation of the immune system. So, it is necessary to identify more precise and effective treatment solutions tailored not only to restore a balanced microbiota, but also to decrease pro-inflammatory markers induced by the abnormal activation of the immune system.

Probiotics, *i.e.* microorganisms recognized for their immunomodulatory properties and role in counteracting dysbiosis have demonstrated the ability to reduce inflammation and improve disease activity in RA patients. However, data on their adverse effects remain scarce, as such events are infrequently reported in randomized controlled trials (168).

Postbiotics, which consist of non-viable microbial products and do not contain live microorganisms, may offer a safer alternative, particularly for immunocompromised individuals (**Table 1**), including RA patients (169). Among postbiotics, EVs released by probiotics are of particular interest, as they can transport bioactive metabolites systemically, reaching distant sites such as the joints. Through cytokine modulation and macrophage polarization, probiotic-derived EVs effectively regulate inflammation, providing a cell-free and safer alternative (150, 170). This favorable safety profile, combined with their capacity to target inflamed joints, highlights probiotic EVs as a promising therapeutic strategy for RA (145, 171).

**Table 1:** Advantages of probiotics and postbiotics, such as probiotic-derived EVs.

Key parameters	Probiotics	Postbiotics (e.g., Probiotic EVs)
Definition	Live beneficial microorganisms	Inactive cells, microbial components or metabolites
Viability	Yes	No
Safety	Rare adverse effects	Safer for immunocompromised patients
Clinical use in RA	Limited	Promising and under preclinical investigation

Despite the lack of studies investigating probiotic EVs effects in RA, some *in vitro* and *in vivo* preclinical models suggest that they are able to suppress inflammation by modulating the immune response. In particular, PFEVs, LJEVs and LPEVs possess anti-osteoclast and anti-inflammatory properties as they can promote M2 macrophage shift and IL-10 secretion (119, 162, 164). Therefore, probiotic EVs might represent the next breakthrough in postbiotics for autoimmune and inflammatory diseases, such as RA, due to their immunomodulatory and anti-inflammatory properties. This phenomenon warrants investigation within the framework of trained immunity, as increasing evidence highlights the capacity of probiotics and their secreted EVs to prime monocytes and macrophages. Considering the pivotal role of these innate immune cells in maintaining and potentially amplifying the inflammatory responses of B and T cells, central players in the pathogenesis of RA, the use of postbiotics as modulators of trained immunity may represent a promising strategy to attenuate chronic inflammation. Moreover, combining probiotic EVs with existing RA treatments, such as biologic or conventional synthetic DMARDs, could enhance therapeutic efficacy by synergistically targeting systemic inflammation and immune dysregulation (172).

### 6.1 Current obstacles and limitations of clinical translation for probiotic-derived EVs

**Figure 5:**
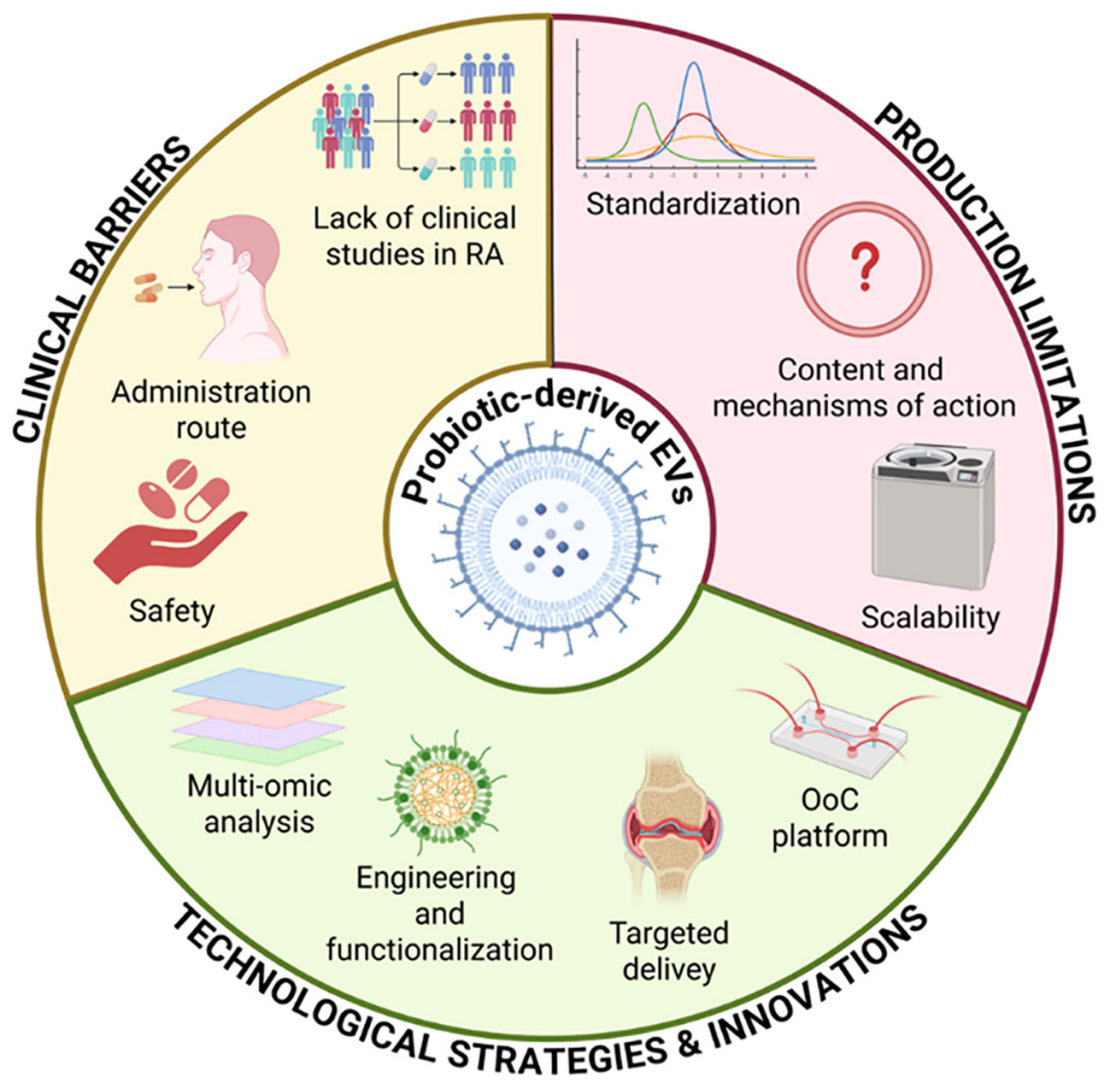
Key factors affecting the clinical translation of probiotic-derived EVs. Standardization, mechanistic insight, and scalability remain major production challenges. Clinical translation is limited by safety concerns, delivery strategies, and lack of studies in RA. Advances in multi-omics, EV engineering, targeted delivery, and organ-on-chip platforms offer promising solutions.

However, before translating this therapeutic approach into a clinical setting (**Figure 5**), the isolation of EVs and the scalability of their production must be standardized. These steps are essential to ensure their clinical safety and reproducibility (123, 173). Even though probiotic-derived EVs exhibit promising immunomodulatory effects, their safety profile must be carefully assessed. Since they are bacterial products, they might still contain substances that can cause immunological reactions, particularly LPS, which makes them potentially pyrogenic and immunogenic if purification is inadequate. Additionally, there is no evidence linking probiotics and their metabolites to allergic reactions. The only reported case regarded the contamination of milk and egg allergens in probiotic compounds (174). In asthma models, for instance, probiotic EVs effects are investigated for their potential to reduce allergic inflammation, with no allergic adverse events reported (175). These findings support the therapeutic potential of probiotic EVs, but they also highlight the necessity of standardized production, purification, and preclinical immunotoxicity testing to guarantee safety.

Advances in omics sciences can provide tools to study probiotic EVs content and mechanisms of action in order to investigate and better understand their potential therapeutic applications. A multi-omics analysis is already used to investigate EVs in different diseases (176) and therefore in this context it might offer detailed insights about their bioactive metabolites.

This approach might be hypothetically applied to enrich, functionalize and engineer probiotic EVs for targeted delivery to inflamed joints. For instance, probiotic EVs can be engineered to enhance their functional properties by physical or chemical strategies to specifically target the bone-gut axis, suggesting a promising therapeutic efficacy (177).

From a clinical perspective, there are currently no registered clinical trials investigating probiotic EVs effects. However, there are few trials exploring the intra-articular injection of EVs, but they are all derived from mesenchymal stem cells (MSCs) and are intended to treat osteoarthritis. MSC-based therapies are being investigated for RA due to their immunomodulatory properties (178), but safety and long-term effects are still under investigation (179). In this context, probiotic EVs emerge as a potentially safer and more practical alternative. The lack of clinical data for probiotic EVs highlights important obstacles like establishing safe and efficient dosage, optimizing delivery routes and demonstrating therapeutic efficacy in RA. Therefore, another challenge that should be faced before a clinical translation it is to standardize the way of administration. EVs can be administered through different strategies, such as local injection, oral ingestion and transdermal delivery (180). Oral administration gives the highest compliance and it is the least invasive option (181). In this regard, orally administered fluorescent EVs in mice have been found also in other organs such as liver, kidney, lung and brain, demonstrating their ability to cross biological barriers (182). So, further research is needed on the interplay of the oral-gut-joint axis since EVs could play an important role in this axis and can offer a new way to study the complex interactions in RA.

### 6.2 Probiotic-derived EVs as a therapy in a patient-tailored approach

To expand further the therapeutic value of these probiotic-derived EVs, more research about engineering them to improve drug loading and specific tissue targeting is needed. For instance, strategies to increase their uptake by tissues and their functionalization to enhance specific anti-inflammatory pathways are currently under investigation (183), which could benefit with the incorporation of the OoC technology.

**Figure 6:**
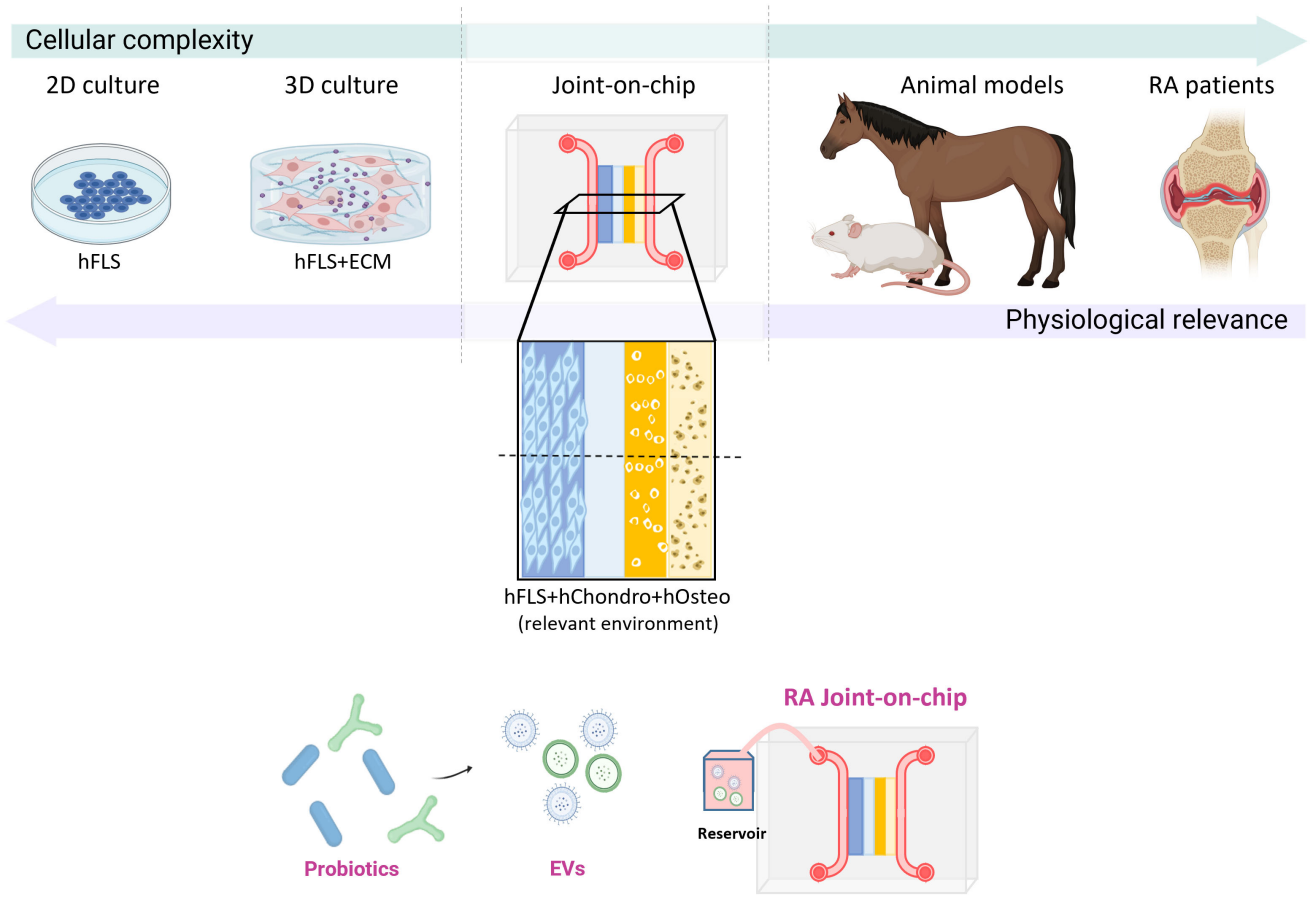
Experimental models to investigate the effects of probiotic-derived EVs in RA. A spectrum of experimental systems is available to model RA pathogenesis, differing in complexity and physiological relevance. These range from 2D and 3D cultures of human synovial fibroblasts to clinical trials involving RA patients, with an incremental increase in physiological fidelity. Animal models provide high biological complexity and disease relevance but are limited by interspecies genetic differences. An advanced joint-on-chip model, incorporating multiple human cell types, including synovial fibroblasts, chondrocytes, and osteoblasts, within a 3D microfluidic platform, would offer a controlled, human-relevant system to evaluate the therapeutic potential of probiotic-derived EVs.

An ideal *in vitro* platform to investigate the immunomodulatory effects of probiotics in RA would require a complex microenvironment that integrates key cellular components of the joint (**Figure 6**). This includes the co-culture of patient-derived synovial fibroblasts (hFLS), osteoblasts, and chondrocytes, in conjunction with autologous immune cells, to closely mimic the cellular crosstalk characteristic of RA pathophysiology. The dynamic administration of bacteria-derived EVs would be essential to model probiotic-host interactions in a physiologically relevant manner (167). OoC models represent a promising technological advance toward this goal, as they allow for precise spatial and temporal control of multi-cellular interactions, mechanical stimulation, and fluid flow, thereby offering a powerful human-relevant platform to dissect the mechanistic impact of probiotic-derived factors on immune modulation and joint inflammation in RA. Nevertheless, the disease complexity of RA could hinder this translation into clinical applications. For this reason, more preclinical studies and clinical trials on probiotic EVs as RA therapy are mandatory.

In conclusion, by addressing these challenges and filling the knowledge gaps, probiotic EVs could help RA treatment, combining their immunomodulatory, gut restoring and drug-delivery properties into a highly biocompatible postbiotic therapeutic approach.
